# Zebrafish as a New Tool in Heart Preservation Research

**DOI:** 10.3390/jcdd8040039

**Published:** 2021-04-08

**Authors:** Luciana Da Silveira Cavalcante, Shannon N. Tessier

**Affiliations:** 1 Center for Engineering in Medicine and Surgery, Massachusetts General Hospital and Harvard Medical School, Boston, MA 2114, USA; ldasilveiracavalcante@mgh.harvard.edu; 2 Shriners Hospitals for Children, Boston, MA 2114, USA

**Keywords:** heart preservation, heart transplantation, regenerative medicine, zebrafish

## Abstract

Heart transplantation became a reality at the end of the 1960s as a life-saving option for patients with end-stage heart failure. Static cold storage (SCS) at 4–6 °C has remained the standard for heart preservation for decades. However, SCS only allows for short-term storage that precludes optimal matching programs, requires emergency surgeries, and results in the unnecessary discard of organs. Among the alternatives seeking to extend ex vivo lifespan and mitigate the shortage of organs are sub-zero or machine perfusion modalities. Sub-zero approaches aim to prolong cold ischemia tolerance by deepening metabolic stasis, while machine perfusion aims to support metabolism through the continuous delivery of oxygen and nutrients. Each of these approaches hold promise; however, complex barriers must be overcome before their potential can be fully realized. We suggest that one barrier facing all experimental efforts to extend ex vivo lifespan are limited research tools. Mammalian models are usually the first choice due to translational aspects, yet experimentation can be restricted by expertise, time, and resources. Instead, there are instances when smaller vertebrate models, like the zebrafish, could fill critical experimental gaps in the field. Taken together, this review provides a summary of the current gold standard for heart preservation as well as new technologies in ex vivo lifespan extension. Furthermore, we describe how existing tools in zebrafish research, including isolated organ, cell specific and functional assays, as well as molecular tools, could complement and elevate heart preservation research.

## 1. Introduction

Several advances in cell therapies and implantable devices have been made in recent years for the treatment of heart disease; however, heart transplantation remains the main recommended treatment for advanced heart failure [1,2]. For every one patient who dies on the heart transplant waitlist, 10 hearts go unutilized from organ donors [3] along with another >30 hearts from potential suitable donors [4,5]. While many of these hearts are not feasible to transplant, many usable hearts nonetheless go untransplanted [5] and complex, interwoven factors play a role in this low utilization.

Integrative solutions are needed to address the organ shortage and extending organ preservation duration has been identified as a priority in transplantation [3,6,7]. Extending the preservation duration of hearts would eliminate circumstantial factors, such as the timing of donor death and recipient/donor location, that contribute to a high rate of discarded organs, reduce the cost of transplantation, convert from emergency to planned surgeries, improve recipient outcomes by matching donors across greater distances, and enable tolerance induction protocols. However, after decades of successful heart transplantations, only modest increases in cardiac preservation duration have been achieved clinically, despite bold new solutions proposed in the literature, including several sub-zero approaches [8,9,10,11,12,13,14]. Additionally, reviving “marginal” organs, especially from non-heart-beating donors, is another promising method [15]. While hearts from donation after circulatory death (DCD) are subject to a longer period of warm ischemia [16,17], they account for most of marginal organs and in some countries, a large, if not majority, of the donor pool [18]. For the heart, researchers conservatively estimate that DCD could increase cardiac donation by 17% [19]. Ex vivo heart perfusion (EVHP) is one of the strategies proposed in the literature to both increase preservation duration and increase the donor pool by resuscitating organs after a warm ischemic insult [20]. However, optimal methods to extend the preservation duration and promote the recovery of DCD hearts using EVHP remain largely unknown. Taken together, despite the advent of innovative technologies in experimental transplantation [21], progress in heart preservation remains hindered, and we suggest that a lack of critical research tools directly undermines these efforts.

Animal models in research are invaluable tools since they allow us to study the kinds of questions that would be impossible with human subjects. Each animal model, from yeast to mammals, has a unique set of advantages (and disadvantages) that enable researchers to strategically address complex scientific questions. *Danio rerio*, the zebrafish, has become a favored research animal as an intermediate model between invertebrate systems (i.e., *C. elegans* and *Drosophila*) and more complex mammalian systems such as mice and rats [22]. Zebrafish has many advantages such as a robust toolbox for genetic manipulation, enabling researchers to rapidly uncover the molecular mechanisms underlying disease. Since about 70% of genes in the zebrafish have corresponding human orthologs, they are becoming important models in broad aspects of human disease [23]. Furthermore, techniques exist to reliably produce transgenic zebrafish on a regular basis, allowing its widespread use in the recent decade [24]. As a result, transgenic zebrafish are powerful tools to directly visualize cell morphology and biology in real-time as a function of treatment. Importantly, all of this can be achieved within the native, 3-dimensional tissue structure in a high throughput format. Finally, zebrafish has been used to study cardiac development and regeneration [25,26], as well as several cardiovascular diseases including cardiomyopathy [27], heart failure [28] and defects in the cardiac conduction system [29], and electrophysiology [30]. Hence, a whole suite of heart-specific tools already exists for immediate application in other fields. 

Taken together, the aim of this review article was to provide a summary of heart transplantation research, especially as it pertains to the extension of preservation duration and EVHP in the context of expansion of the donor pool, and the tools in zebrafish research that could be used to help advance these aspects of heart transplantation research. 

## 2. Current Status in Heart Preservation Research 

### 2.1. Gold Standard in Heart Preservation 

The first human heart transplant was performed in 1967 by Dr. Christiaan Barnard and his team in Cape Town, South Africa [31,32,33]. During this time, the donor and recipient were in the same hospital and hypothermic perfusion was applied. The heart was bypassed and cooled down to 26 °C and subsequently to 16 °C, then finally excised and placed in Ringer’s lactate solution at 10 °C while transported to the adjacent operating room and connected to a coronary perfusion line [31]. This mode of transplantation and preservation technique was used in the subsequent decade and limited total ischemic time to less than 1 h [34] but required the donor and recipient to be in the same hospital at the same time. At the time of retrieval, the donor had shown no spontaneous respiratory movement and no activity in the electrocardiogram for 5 min, therefore characterizing it as a DCD heart [31,35].

Ten years after the first human heart transplant was performed, Thomas and colleagues [34] reported for the first time the long-distance transportation of human hearts for transplantation. They reported six transplants in 1977 where the donor hearts were in different cities and had to be transported to Richmond, VA. In all cases, hearts were transported while immersed in a cold saline solution at 4 °C with ischemic times all under 4 h (minimum observed: 2 h 40 min; maximum observed: 3 h 50 min). The authors concluded that the performance of the hearts was not significantly affected and therefore transportation was feasible [34]. 

Over forty years later, SCS remains the gold standard for heart preservation (and organs in general) for transplantation purposes [36] allowing for inter-city and even inter-state transportation. SCS allows for the preservation of organs for several hours, with hearts being the organs with one of the shortest shelf-lives (4 to 6 h). The low temperature decreases metabolism and consequently the requirement for oxygen, which makes it possible for the organ to survive a few hours without active perfusion. This shelf-life is correlated with how much ischemic injury the organ can endure and it has been shown that short-term and long-term organ function are negatively affected [37] as well as the recipient’s mortality exponentially increases by prolonged cold ischemic times [38,39,40]. 

Currently, the retrieval and storage of hearts for transplant involve the administration of a cold cardioplegic solution through the aortic root for the cessation of heartbeat followed by quick excision and immersion in a bag of 1–2 L crystalloid preservation solution (as a replacement for saline solution in the early days) and placed on ice or cooler for the duration of storage up until it reaches the recipient [41]. The development of preservation solutions has been the only additional improvement in SCS since the late 1970s. The preservation solutions are classified as intracellular or extracellular based on the sodium and potassium contents, which either mimic that of the intra (low Na^+^, high K^+^) or extracellular (high Na^+^, low K^+^) milieu [42]. There are several variations of these preservation solutions with the most used ones for hearts being the University of Wisconsin (UW) solution, Celsior and histidine–tryptophan–ketoglutarate (HTK) [41,42]. The solutions’ composition varies but overall, in addition to sodium and potassium, they also contain an impermeant or colloid that aims to tackle cellular edema, a buffer to maintain pH as cellular metabolites accumulate in the solution and therefore avoiding acidosis, and antioxidants aim to protect cells from reactive oxygen species [36,42,43] as summarized in Table 1. 

### 2.2. Barriers and New Technologies in Heart Preservation

The first heart transplant utilized hypothermic perfusion to continuously perfuse the heart while in the donor then later transferred to a heart lung bypass machine while awaiting implantation in the recipient [31]. Seventeen years after performing the first heart transplant, Barnard and colleagues reported on the development of a portable hypothermic perfusion device to keep hearts preserved for heterotopic heart transplants. They reported on four preserved hearts perfused for 6–15 h with an oxygenated perfusate containing sodium and calcium chloride, potassium phosphate and sodium bicarbonate [45]. Perhaps the portability of the system was not enough to allow for transportation, but the system did not become widely used and since SCS seemed to achieve similar results, it became the standard. 

Several decades later, while looking for better ways to increase shelf-life and increase the donor pool, two seemingly divergent strategies are beginning to emerge in the literature, including sub-zero and machine perfusion modalities. For example, supercooling preservation is designed to push the boundaries of SCS at +4 °C by descending into lower storage temperatures (~−4 to −8 °C). This enables deeper metabolic stasis and a tripling of preservation duration while retaining the preservation media in the liquid state [46]. The potential of supercooling to prolong cold ischemia time has been demonstrated in the liver [9,13] and the heart [14]. However, the supercooled state is thermodynamically unstable, meaning every degree decrease in temperature increases the risk of accidental ice formation, thereby limiting the depth of metabolic stasis that can be achieved. To further prolong cold ischemia time beyond supercooling preservation, others are suggesting an approach dubbed “partial freezing” that mimics freeze-tolerant wood frogs in nature [47] and aims to store whole organs at ~−20 °C for weeks [8,12,48]. However, partial freezing stores organs in the presence of ice, which can be exceptionally damaging if not properly controlled. Others are also exploring deep cryogenic preservation methods using vitrification (<−140 °C), which is the transformation of a substance into a glass through extremely rapid freezing [49,50]. While vitrification holds the promise of achieving long-term banking on the order of years, a requirement for extremely high cooling/heating rates have limited its scalability to whole organ systems.

In contrast, machine perfusion seeks to extend preservation duration through metabolic support at higher temperatures. Perfusion technologies provide a means to continuously deliver oxygen and essential nutrients while also removing metabolic byproducts [20]. Using EVHP hearts can be maintained for several hours and allograft function can be assessed [51]. Machine perfusion may be a particularly good option for retrieving organs that might have gone unused otherwise since it provides a platform to administer pharmacological agents to improve cardiac recovery and function. 

The Organ Care System (OCS™ Heart), a portable normothermic myocardial perfusion device, is in use in transplant centers in Europe, Canada and Australia and is currently under clinical investigation awaiting FDA approval for utilized and marginal donor hearts [52]. The system allows for the extension of the preservation time to 8 h, while allowing for a thorough assessment of the organ before transplantation [53]. Several other normothermic and hypothermic machine perfusion devices have been developed and are either in early development stages or in clinical trials pending FDA approval. While perfusion may provide the moderate extensions of preservation time and unrivalled capacity for organ assessment and/or repair, maintaining ex vivo homeostasis becomes exponentially more complex with increasing perfusion durations and requires continuous monitoring and adaptations to the system [54,55]. Furthermore, optimal EVHP perfusate composition is largely unknown [21], especially as it pertains to initiating the recovery process required to more safely utilize marginal hearts. As a result, the application of EVHP has been limited in the clinical setting, as compared to other organs such as the lung [56].

## 3. The Zebrafish: Our Solution to Overcome Barriers in Heart Preservation Research

**Figure 1:**
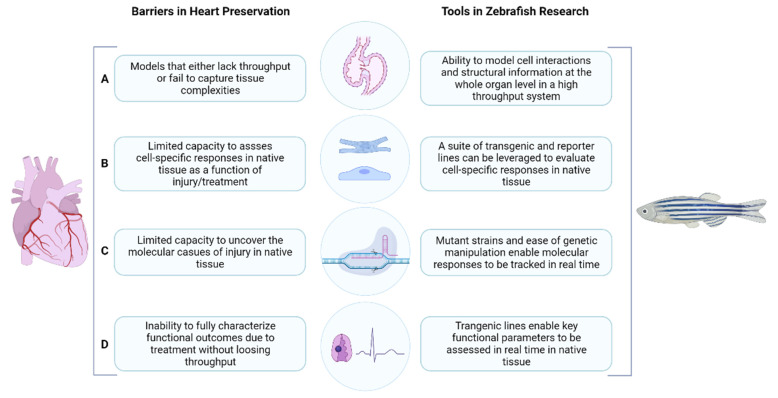
Summary of tools in zebrafish research and how they can help overcome barriers in heart preservation research. Isolated organ assessments (**A**); cell specific assessments (**B**); molecular assessments (**C**); functional assessments (**D**). Created with BioRender.com (accessed on 16 February 2021).

While each of these bold new solutions to extend preservation duration and revive marginal hearts faces unique scientific challenges, they are all connected by a lack of research tools. We propose that this lack of research tools has a direct impact on heart preservation/transplantation research in four important ways, as outlined in Figure 1, and that the zebrafish is a powerful model system which could be leveraged to address these needs. 

Firstly, research efforts in experimental transplantation must consider the tradeoff between throughput and translational potential, often sacrificing one for the other (Figure 1A). While in vitro models like primary cell culture offer throughput, they fail to capture the complexities of tissues. Therefore, the results obtained from those models are less likely to translate into solid organs. On the other hand, the costs and complexity associated with small and large mammalian models are generally high and limits their use for screening purposes. In our view, zebrafish can provide the balance needed by direct access to native whole hearts, while being a much cheaper model to maintain, thereby allowing for higher throughput experimentation. Indeed, zebrafish larvae have been widely used for small molecule and toxicity screening [57], allowing to screen hundreds of conditions while immediately providing information on possible good candidates that allow for the quick reiteration of the screening process in a more effective way. In addition to screening in zebrafish larvae, excised adult zebrafish hearts can be maintained in culture and used as a relatively high throughout platform to mimic many aspects of ex vivo handling for heart transplantation research. These and other examples are described further in Section 4.1 Isolated Organ and Transplantation Assays.

In addition to the screening potential of zebrafish, the type of information that can be gleaned using zebrafish as an experimental platform is also of tremendous value. Other significant challenges with heart preservation and transplantation research are that cardiac tissue is composed of diverse cell types (Figure 1B), each with their own underlying molecular profile (Figure 1C), that interact to perform highly complex functions (Figure 1D). Capturing complex cell/molecular information and still being able to assess functional outcomes is difficult to model, yet all these principles are important in designing new preservation approaches. For example, cardiomyocytes and endothelial cells may have different responses to the same treatment, and these responses are best addressed in intact tissue since it has 3D structural considerations, such as cell–cell and cell–scaffold interactions, that have a profound effect on outcomes. Additionally, the sequence of cellular processes that originate in specialized cells in the atrium and travel all the way to the ventricle, triggering muscle cells to contract and culminating in a heartbeat, is one example of a critical functional assessment that is often lost in simplified experimental model systems. Zebrafish offer a means to interrogate the underlying cell and molecular effects of preservation or warm ischemic injury using a suite of already existing transgenic, reporter, and mutant strains, as well as a robust toolbox for genetic/molecular manipulation. As a result, researchers can track cell and molecular responses as a function of injury, identify causation, and use this knowledge to identify new strategies or therapeutics to overcome injury. Importantly, this can all be achieved within the context of a whole organ system with highly sophisticated downstream assessment tools. We describe specific examples of tools and assays that enable analysis of cell-specific and molecular responses, as well as comprehensive downstream assessment tools in Section 4.2 Cell Specific Assays, Section 4.3 Molecular Assays, and Section 4.4 Functional Assays, respectively. 

Despite the many advantages of the zebrafish model, like any other animal model used in biomedical research there are always limitations. For example, it should be noted that the zebrafish is a small, cold-blooded organism that can survive larger temperature fluctuations, as compared to mammalian cells. Moreover, zebrafish hearts are much smaller, consist of two-chambers, and have regenerative capacity. There are also other important experimental considerations—for example, the presence of duplicate genes can complicate genetic analysis [58]. Nonetheless, provided research is conducted with careful consideration of these limitations and used strategically in combination with more established models in experimental heart transplantation, zebrafish may serve to bridge a critical gap in heart preservation research. In another review, we discuss more broadly the zebrafish approach for organ transplantation research [59], while here we focus exclusively on heart preservation research. 

## 4. Tools in Zebrafish Research 

The main challenge in finding an ideal model for organ preservation research is achieving a good balance among 1—capturing the complexities of whole organ structure; 2—costs/number of animals needed/high throughput; and 3—the outputs that can be measured. Additionally, the feedback loop and ability to learn from past experiments and immediately apply them to current experimentation can help move research along quicker. In this section, we highlight some of the tools available in zebrafish research that could benefit heart preservation research, including isolated organ and transplantation assays, cell specific assays and numerous transgenic, mutant, and reporter lines, as well as functional and molecular assays that allow to study many important pathways in transplantation that can be carried out in the larval and adult stages. It is important to emphasize that the examples provided below are only a snapshot of some of zebrafish tools/assays that could be applied to heart preservation research. 

### 4.1. Isolated Organ and Transplantation Assays

Although the use of zebrafish hearts for ex vivo assays still has not been fully explored, here we provide a few examples of findings in the literature that could be directly applied to heart preservation research. These isolated hearts have the capacity to mimic many of the steps that human hearts go through during ex vivo handling between retrieval and reimplantation.

Pieperhoff and colleagues [60] have reported a zebrafish heart on a plate assay, where the adult heart was excised and kept in culture for 5 days, with functional and histological assessments being performed. They showed that is possible to maintain the heart in culture and spontaneously beating without pacing for up to 3 days. After that period, the heart begins to deteriorate, showing high levels of apoptosis, loss of sarcomere patterns and ventricle wall movement. This type of assay allows for the retrieval of adult hearts for studies involving storage conditions, cocktail formulations and drug screenings that could immensely benefit the preservation and transplantation fields. 

A partial heart transplantation protocol has also been developed by González-Rosa et al. [61] using different transgenic lines and wild type animals that allows for differentiation between host and graft tissues. While the focus of the study was on heart regeneration, it also provides important information and techniques that could easily be applied to studies focusing on heart transplantation, including 30-day post-transplantation survival and procedures and controls that contribute to uptake of the graft like irradiation and cryoinjury. This study helps consolidate the feasibility of a heart transplant assay in zebrafish that could be used to evaluate new preservation and recovery strategies, among others.

### 4.2. Cell-Specific Assays

While we have mastered the preservation of several clinically relevant cell types, what works for a single cell type in suspension often fails to translate to more complex tissues and solid organs. This is because different cell types will have different responses to the same injury and intact tissue has 3D structural considerations that influence outcomes. As such, tools that enable the real-time tracking of specific cells in their native, whole organ structure would allow us to discover cell-specific injury mechanisms occurring during perfusion, cold storage and/or ischemia without losing throughput. In this section, we highlight some examples of cell-specific assays that have been generated in zebrafish that could be performed using microscopy or flow cytometry, either relying on the cell-specific fluorophores of the transgenic lines alone or in combination with immunohistochemistry and other fluorescent dyes. Other advantages include crossing different transgenic fluorescent lines to produce double-fluorescent lines that mark different structures in the heart. Table 2 summarizes some useful fluorescent reporter lines that could be employed in heart preservation/transplantation research. 

Endothelial cells are essential for normal heart homeostasis, in addition to having a particular sensitivity to ischemia–reperfusion [78] and the degree of endothelial injury has been shown to correlate with the functional impairment of the graft following transplantation [79]. Patra and colleagues [62] evaluated endothelial cell distribution in adult zebrafish hearts using several transgenic lines (*Tg(fli1a:EGFP)^y1^*, *Tg(kdrl:Hsa.HRAS-mCherry)^s896^*, *Tg(kdrl:EGFP)*, *Tg(myl7:GFP)^twu34^*). Some of the assays included coronary vessel distribution in *Tg(kdrl:Hsa.HRAS-mCherry)^s896^* immunostained for mCherry and α-actinin/with Alexa-488 conjugated phalloidin to stain cardiac tissue; live/dead cell analysis by FACS using cells from *Tg(kdrl:EGFP)^s843^* and *Tg(myl7:GFP)^twu34^* ventricles stained with DRAQ5™ and 7-aminoactinomycin D (7AAD); the isolation and culture of cardiac endothelial cells for subsequent use in an in vitro wound healing assay. In this study, different transgenic lines were crossed to obtain a line that specifically marks all cardiac endothelial cells and a protocol to isolate these cells was established. The lines described in this study could be used to assess the effects of different preservation/perfusion solutions on endothelial cells. 

In response to hypoxia and/or tissue injury, cardiac fibroblasts rapidly transition to an activated cell type that synthesizes abundant extracellular matrix (ECM) proteins, mediating wound contracture and cell-communication [80]. Resident cardiac fibroblasts are the principal source of activated fibroblasts in mammals and zebrafish in response to injury [81]. A study by Sánchez-Iranzo and colleagues [68] crossed *Tg(−6.8 kbwt1a:GFP)* and *Tg(col1a2:LOXP-mCherry-NTR)* lines to produce a double-transgenic line and evaluate the contribution of fibroblasts to the deposition of the extracellular matrix in hearts undergoing cryoinjury, as well as a line that marks activated fibroblasts *Tg(postnb:citrine)* that appear in response to the injury. The localized cryoinjury is induced with a liquid nitrogen cooled copper probe to damage the ventricle in a manner which mimics myocardial infarction [82]. The study also transplanted kidney-marrow-derived cells from a fluorescent reporter line into wild types and concluded they do not play a role in cardiac fibrosis. The study used confocal microscopy for immunofluorescence imagining and quantitative RT-PCR and RNA-Seq for gene expression analysis. They investigated the origins of activated fibroblasts and tracked their fate using genetic tools to mark periostin b- and collagen 1alpha2-expressing cells, as well as transcriptome analysis. Considering fibroblasts are an important cell type in tissue repair, they may possibly play a role in the homeostasis of the organ/tissue post-transplant and maybe the recovery of marginal organs. Therefore, they are a cell type of interest that should be considered in preservation studies. 

Finally, there are several transgenic lines with fluorescently marked cardiomyocytes in the plasma membrane or nucleus, and several combinations of green and red fluorescence that can be used to determine viability during preservation studies. Here, we highlight a photoconvertible line that could be helpful in transplantation studies. The use of photoconvertible fluorescent proteins in creating transgenic reporter lines has also been widely used for different cells types, including cardiomyocytes, and is a versatile tool. A study by Itou et al. [64] tracking the migration of cardiomyocytes during regeneration used the photoconvertible *Tg3(myl7:Kaede)* line. Kaede is a green fluorescent protein which, once irradiated with UV light, produces a stable red fluorescence, therefore a restricted area of the ventricle was labeled by the red fluorescence while the remaining part (not exposed to UV) continued to exhibit green fluorescence, thus allowing to trace the migration of red-labeled cells during regeneration. The injury method used in the study involved ventricle amputation at the apex. The amputation induced the expression of *cxcl12a* and *cxcr4b* genes in epicardial tissue and cardiomyocytes, respectively. Using the photoconvertible line to track migration in combination with the pharmacological blocking of *cxcr4b* they concluded that the migration of cardiomyocytes to the injury site is regulated by *cxcr4b* and is independent of proliferation. Similarly, a Kaede heart could be irradiated (red), preserved and transplanted into a non-treated recipient (green) allowing to track the transplanted cells and avoid rejection. Otherwise, these studies could be carried out with two different transgenic lines, a mix of wild type and transgenic lines, or using the immunosuppressed line *rag2/c-myb^I181N^* and clonal lines (CG1) [83]. 

On the Zebrafish Information Network (ZFIN) database, there are more than 270 transgenic reporter lines using the myosin light chain 7 (*myl7*, previously known as *cmlc2*) gene as the regulatory region with expression anatomy in the heart and related structures with different options of fluorophores available (i.e., EGPF, mCherry, RFP, etc.). 

### 4.3. Molecular Assays

Another aspect of genetic modifications in addition to transgenic lines and fluorescent reporters is the ability to knock-in or knock-out genes of interest to study their role in healthy and abnormal phenotypes. For transplantation research, this is of particular interest to understand the pathways of organ injury that result in the suboptimal utilization of organs and adverse outcomes post-transplantation, including those caused by ischemia/reperfusion, and low temperature. Here, we discuss only a couple examples of mutant and reporter strains that could be used to uncover the underlying molecular causes of injury during the ex vivo handling of hearts for transplantation. 

The transcription factor hypoxia inducible factor 1 (*HIF-1*) is responsible for adaptations to hypoxia at a molecular level and has been reported to protect several organs, including hearts, from ischemia–reperfusion injury in animal models [84]. Several mutant zebrafish lines have been created including *HIF-1α^−/−^* knockouts and other *HIF-1* isoforms like *HIF-1β^−/−^* knockouts and *HIF-1α^−/−^β^−/−^* double knockouts that were used by Mandic et al. to investigate the hypoxic ventilatory response in zebrafish [85]. A hypoxia reporter line that reflects HIF activity was also developed by Santhakumar et al. and used as an in vivo tool for studying hypoxic signaling in tumors [77]. Work has also been done to evaluate the impact of hypoxia on heart regeneration. Jopling et al. generated a transgenic line that conditionally and specifically express dominant negative *HIF-1α* in cardiomyocytes using the Cre/tamoxifen system. The line was generated by crossing the *Tg(cmlc2a:Ert2-Cre-Ert2/cmlc2a:LnL:GFP)* line with a *Tg(cmlc2a:LrL:dnHIF1α)* line which contains a floxed red fluorescent protein stop cassette (*LrL*). The treatment of embryos or adult zebrafish with 4-hydroxytamoxifen results in a recombination of the floxed stop cassette and subsequently a rapid, cardiomyocyte-specific induction of dominant negative *HIF1α*. They were able to show that hypoxia has a positive regulatory effect on heart regeneration [86]. 

Another protein family of interest is heat-shock proteins that have long been identified as contributing to cell survival during stress conditions. Among those, we highlight *HSP70* and *HSP90*, which are implicated in low temperature stress responses. A mutant knockout line of *HSP90* has been described [87], but there are not many reports of stable mutant lines with most genetic manipulation being done with morpholinos at the embryonic stage. Morpholino is an oligomer molecule utilized to modify gene expression by inhibiting the translation of RNA transcripts in vivo. It is widely used in zebrafish as a standard knockdown tool in early embryonic stages [88]. The same is true for *HSP70* knockouts, although one could still achieve inhibition and/or overexpression using environmental and pharmacological treatments. An *HSP70* reporter line *Tg(hsp70l:EGFP)* has been generated and is widely used in toxicological studies [89,90].

### 4.4. Functional Assays

When testing new preservation protocols or perfusion/cardioplegic solutions, the in-depth analysis of tissue functionality is crucial. While individual cell assays are a great starting point to screen-promising conditions, cell viability does not necessarily translate to functionality. The zebrafish model also allows for functional assays and in this section, we will summarize a few tools for heart transplantation research. 

A fluorescent cardiac-specific Ca^2+^ indicator transgenic line *Tg*(*cmlc2*:*gCaMP*)*^s878^* was described by Chi et al. [69] to analyze the formation of the cardiac conduction system. Calcium is the major molecule in cell signaling, controlling almost every aspect of cellular physiology. In transplantation research, this line could be used to assess calcium homeostasis and signaling in cardiomyocytes, allowing for an evaluation of cardiac conduction and excitability and to detect any disruptions to the system that could potentially lead to arrhythmias. In addition, the expression of gap junction proteins *Cx40* and *Cx43* responsible for cardiac conduction can also be analyzed by immunostaining [69]. The electrical activity of the whole heart can also be recorded using electrocardiography (ECG) according to several reports [91,92,93]. 

Using immunohistochemistry, it is possible to stain the main extracellular matrix proteins in zebrafish hearts: fibronectin, collagen, and fibrinogen [94] to gather more information about structural integrity, but for functional assessments, is also possible to use a transgenic line to evaluate myofibril structure. Myofibril structure and precise assembly is directly related to the contractile performance of the heart [95]. Reischauer and colleagues generated the transgenic line *Tg(myl7:LifeAct-GFP),* in which filamentous actin (a component of sarcomeres) is labeled with GFP [70]. Using this line combined with confocal microscopy, it is possible to analyze cardiomyocyte contractility and remodeling as well as distinguish the architectural differences in myofibril organization in cardiomyocyte subtypes (atrium, ventricular wall, and ventricular trabeculae). The authors also used this line to show the evolution of myofibril arrangement during cardiac development and evaluated the effect of *Erbb2* signaling (a target for many cancer drugs) in myofibril remodeling since these drugs can cause cardiomyopathy in many patients [70]. In a similar manner, this line could be used to assess the effects of preservation on the structural integrity of the heart. 

Barrier function is an important role of endothelial cells. An in vitro vascular permeability assay using fluorescein isothiocyanate (FITC)-conjugated dextran (70 kDa, FITC-dextran) has been previously described by Pauty et al. [96] using a human microvessel 3D model. In our lab, we adapted this assay by combining it with a transgenic line *Tg(fli1a:mCherry)*, which marks endothelial cells with red fluorescence, to assess the effects of preservation on vascular permeability in zebrafish larvae. 

## 5. Conclusions

Apart from portable perfusion devices, very few advances have been made in heart preservation in recent decades. Meanwhile, in the past two decades, the zebrafish has become a powerful model to study cardiac development, regeneration, and disease. The fact that it is a fish, with size constraints and regenerative capacities that are not observed in mammals, may constitute a limiting or discouraging factor for many. There are also anatomical considerations like the presence of only two non-septated heart chambers, and morphological differences in the valves [58]. However, if used strategically in combination with mammalian models, it can offer complimentary tools that would not be feasible otherwise. The ease of genetic manipulation combined with fast development and the possibility of real-time imaging of an in vivo processes, the number of cell specific and functional assays, the ability to culture hearts ex vivo for storage studies and perform partial heart transplants make zebrafish a valuable and innovative candidate to help advance heart transplantation research. 

## Figures and Tables

**Table 1:** Clinically used heart preservation solutions.

Solutions	University of Wisconsin Solution	Celsior^®^	Custodiol^®^ HTK Solution
Type	Intracellular	Extracellular	Extracellular
Buffer	Phosphate	Histidine	Histidine
Antioxidant	Glutathione, Allopurinol	Glutathione, Mannitol	Mannitol
Colloid	Hydroxyethyl starch, Lactobionic acid, Raffinose	Mannitol, Lactobionic acid	Mannitol
Intended use	Flushing/sold storage	Flushing/cold storage	Flushing/cold storage
Other	High viscosity and tendency of particle formation (might require filtration)	Combines elements of UW and HTK	Lower viscosity and potassium compared to UW
References	[42,44]	[42,43,44]	[42,44]

UW: University of Wisconsin; HTK: histidine–tryptophan–ketoglutarate.

**Table 2:** Fluorescent reporter lines with expression anatomy in the cardiovascular system and other systems of interest for heart transplantation research.

Regulatory Region	Cardiovascular Structure	Construct	References
*kdrl*	Cardiac endothelial cells/endocardium	*Tg(kdrl:EGFP) (cytosolic)* *Tg(kdrl:Hsa.HRAS-mCherry) (plasma membrane)*	[62,63]
*myl7*	Cardiomyocytes	*Tg(myl7:GFP)*	[62]
*myl7*	Cardiomyocytes	*Tg3(myl7:Kaede)*	[64]
*myl7*	Nucleus of cardiomyocytes	*Tg(myl7:h2afva-mCherry)^sd12^*	[65]
*myl7*	Endocardial cells	*Tg(Tie2:EGFP)*	[66]
*tcf21*	Epicardial cells	*Tg(tcf21:DsRed2)*	[67]
*wt1a*	Cardiac fibroblasts	*Tg(−6.8kbwt1a:GFP)*	[68]
*col1a2*	Collagen producing cells	*Tg(col1a2:LOXP-mCherry-NTR)*	[68]
*postn*	Perivascular and epicardial cells (uninjured hearts), activated fibroblasts (injured hearts)	*Tg(postnb:citrine)*	[68]
*myh6*	atrial cells	*Tg(myh6:EGFP)^s958^*	[65]
*myl7* (previous name *cmlc2*)	Cardiac specific calcium indicator	*Tg(myl7:GCaMP)*	[69]
*myl7* (previous name *cmlc2*)	Cardiac filamentous actin (F-actin)	*Tg(myl7:LifeAct-GFP)*	[70]
*fli1a*	Endothelial filamentous actin (F-actin)	*Tg(fli1a:LifeAct-mClover)*	[62]
*acta2*	Mural cell α-smooth muscle actin	*Tg(acta2:EGFP)* *Tg(acta2:mCherry)*	[71]
*gata1a*	Blood cells (erythroid)	*Tg(gata1:DsRed) (progenitor cells)* *Tg(gata1:DsRed;globin:GFP) (mature erythrocytes)*	[72]
*mpo/lyz* (previous name *lysC*)	Blood cells (leukocytes I) Double transgene (neutrophils and macrophages)	*Tg(MPO:EGFP) × Tg(LysC:DsRed)*	[73]
*mhc2dab*	Blood cells (leukocytes II) B lymphocytes, eosinophils, Macrophages and dendritic cells	*Tg(mhc2dab:GFP)*	[74]
*cd45*	Blood cells (leukocytes III) Myeloid cells and T Lymphocytes	*Tg(cd45:DsRed)*	[74]
*foxp3a*	Regulatory T cells	*TgBAC*(*foxp3a*:*TagRFP*)^vcc3^	[75]
*fli1a*	Vasculature/endothelial cells	*Tg(fli1a:Myr-mCherry)*	[76]
*pdgfrb*	Vasculature/mural cells	*TgBAC(pdgfrb:EGFP)*, *TgBAC(pdgfrb:mCherry)*	[76]
*egln3* (previous name *phd3* )	Hypoxia	*TgBAC(egln3:EGFP)*	[77]

*kdrl*: kinase insert domain receptor like; *myl7*: myosin light chain 7; *tcf21*: transcription factor 21; *wt1a*: wt1 transcription factor a; *col1a2*: collagen type 1 alpha 2 chain; *postn*: periostin; *myh6*: myosin heavy chain 6; *cmlc2*: cardiac myosin light chain 2; *fli1a*: friend leukemia integration factor 1a; *acta2*: actin alpha 2; *gata1a*: GATA binding protein 1a; *mpo*: myeloperoxidase; *lyz (lysC)*: lysozyme; *mhc2dab*: major histocompatibility complex class II DAB; *cd45*: leukocyte common antigen; *foxp3a*: forkhead box P3a; *pdgfrb:* platelet derived growth factor receptor beta; *egln3:* egl-9 family hypoxia inducible factor 3; *phd3*: prolyl hydroxylase domain-containing protein 3.
